# STING, a critical contributor to SARS-CoV-2 immunopathology

**DOI:** 10.1038/s41392-022-00967-3

**Published:** 2022-03-30

**Authors:** Heyu Li, Fangfang Zhou, Long Zhang

**Affiliations:** 1 School of Medicine, Zhejiang University City College, 310015 Hangzhou, Zhejiang China; 2 Institutes of Biology and Medical Science, Soochow University, 215123 Suzhou, China; 3 MOE Laboratory of Biosystems Homeostasis and Protection and Innovation Center for Cell Signaling Network, Life Sciences Institute, Zhejiang University, 310058 Hangzhou, China

**Keywords:** Innate immunity, Infection

**Fig. 1:**
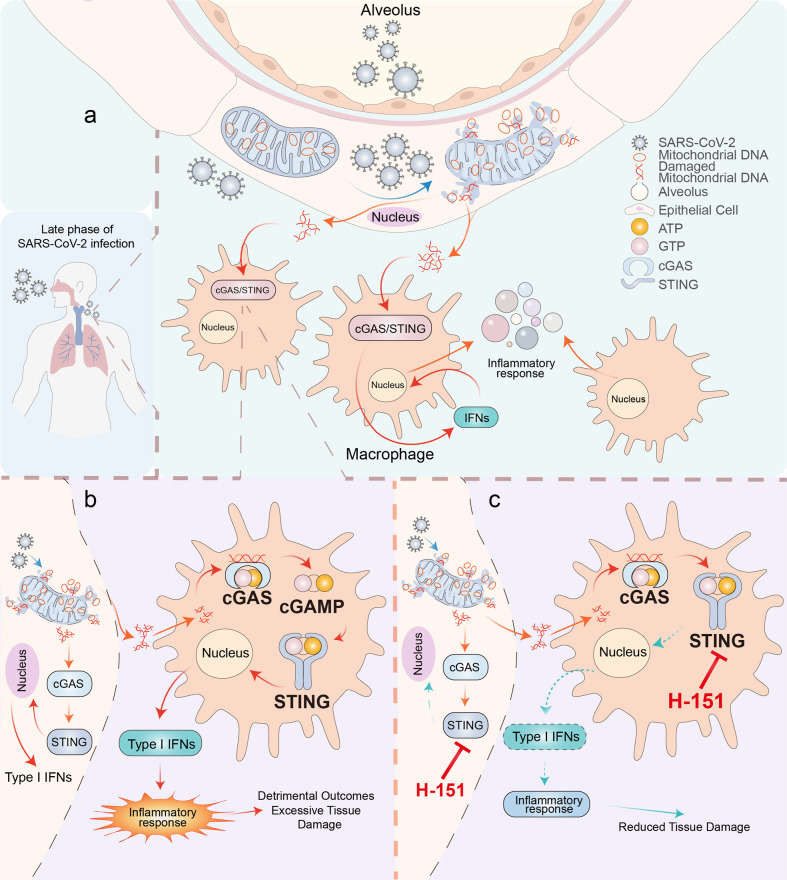
The contributing role of STING in inflammatory responses during the late phase of SARS-CoV-2 infection. **a** The late phase of SARS-CoV-2 infection causes mitochondrial damage in epithelial cells, resulting in the accumulation of mitochondrial DNA (mtDNA) in the cytosol. Furthermore, large amounts of mtDNA activate the cGAS-STING pathway excessively, leading to the death of epithelial cells. When macrophages engulf the dead cells debris at the vessel lesions, mtDNA triggers cGAS-STING signaling. As a result, macrophages produce a large amount of type 1 interferon (IFN), causing a robust inflammatory response. The dysregulated cytokine responses drive immunopathology, including tissue damage and acute body harm. **b**, **c** STING plays a central role in inflammation, and the small-molecule STING inhibitor H-151 exerts great effects in relieving the immunopathology response and ameliorating the lung pathology at the late stage of SARS-CoV-2 infection

Recently, a study published in *Nature* by Domizio et al.^1^ revealed the underlying mechanism of aberrant immunopathology during the late phase of infection with severe acute respiratory syndrome coronavirus 2 (SARS-CoV-2). The authors provided evidence demonstrating that stimulator of interferon genes (STING) plays a unique role in the alveolus-capillary cytokine storm induced by mitochondrial DNA (mtDNA) during coronavirus disease 2019 (COVID-19) (Fig. 1). Some studies stressed that the STING agonists block the SARS-CoV-2 infection via triggering the type I interferons (IFNs) response,^2,3^ while others revealed that SARS-CoV-2 induces the chromatin DNA traveling to cytosol by promoting the cell-to-cell fusion, resulting in the activation of cGAS-STING signaling.^4^ This paper provides a new perspective regarding the role of cyclic GMP-AMP synthase (cGAS)-STING pathway in pulmonary inflammation during SARS-CoV-2 infection.

The COVID-19 pandemic has caused a dramatic loss of life and slowed the global economy. It has been reported that type I IFNs considerably impact SARS-CoV-2 infection, and the effect of type I IFNs depends on their dosage. Continuous high levels of type I IFNs drive the immunopathology in the late phase of infection. Therefore, it is vital to appropriately modulate the type I IFN signaling pathway during the COVID-19 treatment. The cGAS-STING pathway is a crucial part of type I IFN responses. Several studies have reported that the cGAS-STING pathway participates in SARS-CoV-2 infection.^2–5^ DiABZI, a STING agonist, effectively blocks SARS-CoV-2 infection by stimulating type I IFN responses.^2,3^ While Rui et al.^5^ revealed that SARS-CoV-2 suppressed the cGAS-STING pathway via ORF3a and 3CL, Zhou et al.^4^ showed that SARS-CoV-2 promotes cell-to-cell fusion via spike protein, leading to chromatin DNA shuttling from the nucleus and triggering the cGAS-STING pathway eventually. It remains to be investigated whether SARS-CoV-2 activates the cGAS-STING pathway via other mechanisms at the alveolus-capillary interface.

Domizio et al.^1,4^ put great efforts into studying the skin lesions during SARS-CoV-2 infection. COVID-19 profiles exhibited aberrant inflammatory response signatures resembling cutaneous lupus erythematosus (CLE) samples. A large number of genes related to macrophage function are upregulated during skin injury. In addition, there was interferon-beta (IFN-β) induction and a higher level of CD163^+^ macrophages in the surrounding injured vessels. These results indicate that SARS-CoV-2 infection drives skin injuries and recruits type I IFN-producing macrophages to the damaged site. Moreover, the authors investigated the mechanism of type I IFN production by macrophages. Remarkably, COVID-19 profiles showed increased cyclic GMP-AMP (cGAMP) and phosphorylated STING (p-STING) levels. In addition, the STING inhibitor H-151 was shown to alleviate inflammatory responses. Further experiments demonstrated that the cGAS-STING pathway is essential for type I IFN responses in skin lesions. Additionally, lung samples with early diffuse alveolar damage (DAD) displayed high levels of p-STING but not late DAD.

The authors utilized a lung-on-chip (LoC) model to mimic the alveolar-capillary interface. In the LoC model, endothelial cells can be effectively infected by SARS-CoV-2 in vitro. Under infection, LoC endothelial cells expressed large amounts of p-STING and produced high levels of IFN-β. Additionally, H-151 reduced inflammatory responses and cell death, consistent with the previous results observed in samples of patients with COVID-19. Subsequently, Domizio et al.^1^ claimed that STING is over-activated in endothelial cells during infection. It is well-known that the cGAS-STING pathway is involved in detecting cytosolic DNA, while SARS-CoV-2 is a positive-sense RNA virus. It was interesting to determine how SARS-CoV-2 triggered the cGAS-STING signaling pathway. To elucidate the central mechanism, Domizio et al.^1^ investigated the differential factors in the cytosol of endothelial cells by mass spectrometry pre- and post-infection. A large number of mitochondrial proteins were enriched after infection. In addition, electron microscopy revealed damaged mitochondria in skin biopsies. Moreover, the authors speculated that SARS-CoV-2 may drive mitochondrial damage, resulting in mtDNA accumulation in the cytosol. Then, the cGAS-STING pathway was triggered by elevated leaking mtDNA. Furthermore, by manipulating mtDNA via 2′3′-dideoxycytidine (ddC) or VBIT-4, the authors confirmed that mtDNA is an inevitable factor during cGAS-STING activation in SARS-CoV-2 infection.

Given the essential role of STING in SARS-CoV-2-induced lung pathology, Domizio et al.^1^ examined whether the inhibition of STING exerts anti-SARS-CoV-2 effects in a K18-hACE2 transgenic mouse model. It was impressive that H-151 treatment reduced lung inflammation compared to control mice 6 days post-infection (dpi) but not at 3 dpi. Actually, H-151 treatment showed therapeutic functions at maximal viral loads by decreasing cytokine responses and reducing tissue damage. Based on the above findings, the authors concluded that STING elicits inflammatory responses in the late phase of infection but not in the early phase.

Domizio et al.^1^ attempted to answer the critical question of whether SARS-CoV-2 causes endothelial cell damage, stimulating the cGAS-STING signaling pathway and driving immunopathology. For the first time, these researchers clarified that SARS-CoV-2 infection promotes mitochondrial damage and mtDNA leakage in endothelial cells. More importantly, the study provided a new perspective regarding SARS-CoV-2 infection and aberrant immune response at the alveolus-capillary interface in the late phase of infection. These findings were of great significance for the in-depth understanding of the cGAS-STING pathway activation during COVID-19 pathology. Additionally, it was the first time that a STING inhibitor proved to have therapeutic effects against SARS-CoV-2.

More importantly, it is of great value to reconsider the therapeutic potential of STING agonist against SARS-CoV-2. Previous studies reported that a STING agonist, diABZI, effectively blocks SARS-CoV-2 infection.^2,3^ Considering the critical role of STING in aberrant cytokine responses in the late phase of infection, it is vital to investigate whether diABZI leads to heave tissue damage or uncontrollable immunopathology or not. On the other hand, the inhibitors of cGAS-STING pathway may exert efficacy against inflammation. H-151, a STING inhibitor, exhibits therapeutic potential as an anti-inflammation agent via alleviating detrimental immune response. H-151 restricts the delayed type I IFN responses during the late phase of infection and protects body against inflammation. In addition, it is promising that cGAS inhibitors may also exhibit the same effect as H-151. Therefore, the choice of cGAS-STING signaling regulators may vary according to the infection stage to obtain satisfactory therapy outcomes in the future.

In summary, Domizio et al.^1^ unveiled the unique contributing role of STING in enhancing inflammatory responses in the late phase of SARS-CoV-2 infection. They identified H-151, a STING inhibitor, which may serve as a therapeutic agent to reduce the detrimental inflammation against SARS-CoV-2 in the future.
